# NFE2L3 promotes malignant behavior and EMT of human hepatocellular carcinoma (HepG2) cells via Wnt/β‑catenin pathway

**DOI:** 10.7150/jca.48100

**Published:** 2020-10-08

**Authors:** Yonggang Ren, Yujiao Wang, Shuai Hao, Yuhan Yang, Wendong Xiong, Lu Qiu, Jia Tao, Aifa Tang

**Affiliations:** 1 Guangdong Key Laboratory of Systems Biology and Synthetic Biology for Urogenital Tumors, Institute of Translational Medicine, First Affiliated Hospital of Shenzhen University, Shenzhen Second People's Hospital, Shenzhen, Guangdong 518035, P.R. China.; 2 Department of Biochemistry, North Sichuan Medical College, Nanchong, Sichuan 637000, P.R. China.; 3 Department of Clinical Medicine, North Sichuan Medical College, Nanchong, Sichuan 637000, P.R. China.; 4 School of Life Sciences, Zhengzhou University, Zhengzhou 450001, P.R. China.; 5 Department of Pathology, Affiliated Hospital of North Sichuan Medical College, Nanchong, Sichuan 637000, P.R. China.

**Keywords:** NFE2L3, Hepatocellular Carcinoma, epithelial mesenchymal transition, β-catenin

## Abstract

**Objective:** NFE2L3 is a member of the cap 'n' collar basic-region leucine zipper family. NFE2L3 has turned out to be associated with oxidative stress, but the relevance of NFE2L3 in hepatocellular carcinoma (HCC) has remained elusive. This study aimed to investigate the role of NFE2L3 in HCC and explore underlying mechanisms.

**Methods:** Quantitative real-time PCR, western blot and immunohistochemistry were used to detect the mRNA and protein expression of NFE2L3, the expression of epithelial-mesenchymal transition (EMT) markers and Wnt/β-catenin signaling pathway-related proteins. In loss-function experiments, HepG2 cells were transfected with lentiviral vector containing NFE2L3 short hairpin RNA or scramble control. Cell proliferation and migration were measured by Cell Counting Kit-8, Colony formation, EdU incorporation and Transwell assays respectively. Flow cytometry was used to analyze cell cycle and apoptosis. HepG2 cells were subcutaneously injected into nude mice and tumor size was measured once every other day.

**Results:** The results revealed that high expression of NFE2L3 was positively associated with malignant behavior and EMT in HCC. Knockdown of NFE2L3 inhibited cell proliferation and migration, led to cell cycle G0/G1 arrest and induction of cell apoptosis, increased expression of E-cadherin and decreased expression of N‑cadherin, Vimentin, MMP2, CDK2 and PCNA. In addition, tumor growth was inhibited by silencing of NFE2L3 *in vivo*. Expression of β-catenin and Wnt target genes cyclin D1 and TCF4 was reduced in HepG2-shNFE2L3 cells.

**Conclusions:** NFE2L3 promotes cell proliferation, metastasis, and induces EMT of hepatocellular carcinoma (HepG2) cells via activation of Wnt/β-catenin pathway.

## Introduction

Hepatocellular carcinoma (HCC) is one of the main malignant tumors that remain a serious threat to human health. As a major hepatic carcinoma country, China has about 370,000 new patients of hepatic carcinoma and about 326,000 deaths each year 1,2. The early symptoms of HCC are not obvious, and the specificity of the initial symptom is so unclear that we cannot find it. HCC usually has a poor prognosis and a low five-year survival rate 3. Hepatitis B, hepatitis C, alcoholism, and diabetes are high-risk factors for HCC 4. HCC is prone to intrahepatic metastasis in the early stage, and it often metastasizes to the lung, bone and other parts through the lymphatic or blood-way in the middle and late stages. And among the various treatment methods for hepatocellular carcinoma, early surgical resection is still the main treatment for HCC, while adjuvant radiotherapy and chemotherapy 5. Therefore, clarifying the disease mechanisms of HCC and exploring the causes of its malignant behavior are significant for the early diagnosis of HCC and would be helpful for developing new gene-targeted drugs.

NFE2L3 (Nuclear factor erythroid 2-like 3), also known as NRF3, was discovered in the 1990s, which is one of the members of the CNC protein family 6. The CNC protein family encompasses NFE2L1, NFE2L2, NFE2L3, NF-E2, Bach1 and Bach2. All of the factors have similar domain, which means that they may exert similar functions in an organism. As the least noticed member of this family, only extremely limited information for NFE2L3 was published, because NFE2L3-deficient mouse did not show significant pathological manifestations under physiological conditions 7,8. However, with the research on the mechanisms of homologous factors NFE2L1 and NFE2L2, NFE2L3 has become a research hotspot project in recent years. NFE2L3 is expressed in various tissues of the human, but different expression levels suggest the functional differences of NFE2L3 in different tissues. The level of NFE2L3 in placental, cornea, bladder is high, and other tissues are converse 9. The homologous NFE2L2 has been proved to be a driver gene of malignant tumor, and it plays an excellent role in regulating oxidative stress 10. NFE2L2 and NFE2L3 have been proved to be important mutant genes of 12 kinds of malignant tumors in genome sequencing work, and the number of related factors is 127 11. The function of NFE2L3 in the development of pancreatic cancer, colorectal cancer and other malignant tumors has been confirmed, and it is related to the occurrence of carcinogen-induced lymphoma in mice 9,12,13. Meanwhile, it is also reported that NFE2L3 plays a protective role in the process of hematopoietic malignancies and breast cancer 14. NFE2L3 is located in the cytoplasm under physiological conditions, and during the occurrence of malignant tumors, it translocates into the nucleus with the help of the aspartic protease DDI2 as a transcription factor to regulate the expression of downstream genes. The maintenance of protein homeostasis is related to the degradation of proteins by 26s and 20s proteasomes dependent on ubiquitination and non-ubiquitination. In the process of malignant tumors, protein homeostasis is often broken, and 20s proteasome-degraded system dominates. POMP is the assembly partner of the 20s proteasome, and NFE2L3 induces the expression of POMP, so NFE2L3 plays a cancer-promoting role through the axis of NFE2L3-POMP-20s, which reflects the prognosis of colorectal cancer directly 11. The survival rate of patients with colorectal cancer is extremely low in those with high expression of NFE2L3. Zhang *et al* showed that the absence of NFE2L3 induces cell cycle arrest at the G0/G1 phase in colorectal cancer cells 15. These findings suggest that NFE2L3 may be a key regulator of cancer progression. Although the physiological and pathological significance of NFE2L3 is coming into sight, the clinical significance and prognostic value of NFE2L3 in HCC patients remain largely unknown and require further investigations.

In our study, we constructed NFE2L3-deficient HepG2 cells, the results showed that the proliferation, migration and tumorigenic ability with low expression of NFE2L3 are weaker than those of control cells, and cell cycle-related proteins CCND1 and CDK2 are decreased. Furthermore, the enhancement of tumor cell migration ability may be achieved by affecting the EMT, which process is dominated by NFE2L3, and knockdown of NFE2L3 inhibits Wnt/β-catenin signaling pathway. Wnt/β-catenin signaling pathway exerts enormous influence on many kinds of carcinoma. When activated, the signaling pathway may change the cell behavior which brings about the disorder of the cell. Subcutaneous tumorigenesis experiments and immunohistochemical also support NFE2L3 as an oncogene whose high expression is involved in tumor cell malignant proliferation. In summary, NFE2L3 is related to the malignant behavior of HCC, and may promote EMT by activating the Wnt/β-catenin pathway.

## Materials and Methods

### Cell culture and clinical samples

Human hepatocellular cancer cell lines (HepG2, MHCC97L, MHCC97H, Hep3B, Huh7) and human normal liver cell HL7702 were purchased from the Type Culture Collection of the Chinese Academy of Sciences (Shanghai, China) and preserved in our laboratory. HL7702 cells were cultured in RPMI-1640 medium (Gibco, USA), HepG2, Hep3B, MHCC97L, MHCC97H and Huh7 cells were cultured in DMEM medium (Gibco, USA). All media were supplemented with 10% fetal bovine serum (Gibco, USA), 1% penicillin/streptomycin (Gibco, USA) and cells were cultured in a 37 °C, as well as 5% CO_2_ humidified incubator.

Hepatocellular cancer tissues and matched adjacent normal tissues were collected from patients admitted at Affiliated Hospital of North Sichuan Medical College. All cancer cases were confirmed as HCC through pathological diagnosis. The study protocol was approved by the local ethics committees (North Sichuan Medical College, Nanchong, China).

### HCC patient data in The Cancer Genome Atlas and NCBI GEO

A total of 371 HCC patients with clinicopathological information and RNA-Seq data were obtained from TCGA (https://tcga-data.nci.nih.gov/) and GSE25097/GSE76427 data were downloaded in NCBI GEO database (https://www.ncbi.nlm.nih.gov/geo/). The gene expression data were normalized using the RNA normalization method as described 16.

### shRNA knockdown NFE2L3

NFE2L3 shRNA interference lentiviral vector was constructed and synthesized by Cyagen (Guangzhou, China). The NFE2L3 shRNA interference target sequences were sh1: 5'- GCATGTTAAGTAGATATTATC-3', sh2: 5'-CGCGTAGACCTAGATCTTTAC-3', and a scramble sequence 5'-CCTAAGGTTAAGTCGCCCTCG-3' were used as a negative control (NC). The lentivirus was transfected into HepG2 cells according to the manufacturer's instructions. The cells were seeded (1×10^5^ cells/ml) into 6-well plates and incubated for 24 h to reach 50%, and then replaced with fresh medium containing 50 μl of the packaged lentiviral supernatant (MOI=10) and 5 μg polybrene. Successfully infected shNFE2L3 cells were red fluorescent positive observed under fluorescence microscope after 72 h, these positive cells were cultured in puromycin medium for 7 days (2.5 μg/ml). The interference efficiency of NFE2L3 was determined using RT-qPCR and western blot.

### Cell counting kit-8 (CCK-8) assay

CCK-8 assay was conducted to determine cell proliferation. Cultured cells were inoculated into 96-well plates at a density of 5000 cells per well, the cell viability was measured every 24 hours using CCK-8 assay kit (Dojindo Laboratories, Japan) following the manufacturer's instructions. OD values at 450 nm were measured by a microplate reader.

### 5-ethynyl-2'-deoxyuridine (EdU) incorporation assay

Briefly, cells were seeded in 96-well plates, then incubated with 50 μM EdU for 2 hours and stained with Apollo^®^ fluorescent dye, according to manufacturer's instructions (Cell-Light EdU Apollo488 *In vitro* Kit, Ribobio, China). Images were acquired under a fluorescent microscope at 488 nm excitation.

### Colony formation assay

For the colony formation assay, 3000 experimental cells were seeded in 60 mm cell dish, and incubated at 37 °C for 2 weeks. Then cells were washed with PBS and fixed with 4% paraformaldehyde, air-dried and stained with 0.1% crystal violet (Solarbio, Beijing, China). Colonies with >50 cells were counted under a microscope.

### Migration assay

Cell migration assay was conducted using a transwell chamber (8-μm pore size, Corning, USA). Experimental cells were suspended in media containing 1% FBS and seeded in the upper chamber, with the lower chamber containing media supplemented with 10% FBS. After incubation for 8 h, cells were fixed with 4% paraformaldehyde, dried and stained with 0.1% crystal violet (Solarbio, Beijing, China) the remaining cells in the upper chamber were gently removed. Cells that had passed through the pore and adhered to the lower membrane surface were counted with five random fields.

### Cell cycle and apoptosis analysis

Cell cycle experimental cells were harvested and washed with PBS, centrifugation to remove supernatant and fixed in 70% ethanol 2 hours at 4 °C. After fixation, cells were washed thrice in PBS, filtered through a 200-mesh nylon membrane, and then incubated 30 min at room temperature with 100 μl RNaseA (Kaiji, Nanjing, China). Finally, cells were stained with propidium iodide (Kaiji, Nanjing, China) for 30 min at 4 °C in the dark. Apoptosis detection cells treated with hydrogen peroxide and washed thrice with PBS, suspended in binding buffer. Mixed with 5 μl Annexin V-APC and 5 μl PI solution for 15 min in the dark. Cell cycle and apoptosis detection were performed using BD Accuri™ C6 Flow Cytometer (Becton-Dickinson, USA).

### Subcutaneous tumor xenografts in nude mice

All animals used in experiments were conducted in accordance with the regulations of the Animal Care and Use Committee of North Sichuan Medical College. Male Balb/c nude mice (4-5 weeks, 18-22 g) were purchased from the Laboratory Animal Center of North Sichuan Medical College (Nanchong, China) and kept at standard animal housing conditions with proper food and water. The xenograft models were made by subcutaneous heterotransplantation of HepG2 cells into nude mice as described 17. Experimental cells (1×10^7^) were inoculated subcutaneously into the right back region of nude mice at a single site. Once the tumor xenografts emerged, their sizes were measured once every other day, the mice were sacrificed before the transplanted tumors were excised. The sizes of growing tumors were calculated by a standard formulate (i.e. V= 0.52×width^2^×length) and shown graphically (n= 5 per group).

### The immunohistochemistry of xenograft and human HCC tissues

Xenograft tumor samples and human HCC tissues were paraformaldehyde (4%)-fixed and sectioned into a series of 4-μm-thick slides. The sections were de-paraffinized in a solution of xylol and dehydrated in the concentration-graded ethanol before inactivation of endogenous peroxidase activity. Subsequently, slides were boiled in microwave for 15 min in a citrate buffer (pH 6.0) to retrieve antigen, and blocked with 5% bovine serum albumin. Next, incubated 4 hours at room temperature with NFE2L3 antibody (LSBio (LS-C430024), 1:30). Thereafter, slides were re-incubated with peroxidase-conjugated secondary antibody at room temperature, and visualized by the DAB kit (Proteintech, Wuhan, China). In similar experimental settings, the negative controls were set up by replacing the primary antibody with the normal non-immune serum diluted in PBS. The resultant images were obtained under a light microscope (Leica DMIRB, Germany) equipped with a DC350F digital camera.

### Quantitative real-time PCR (RT-qPCR)

Total RNA was extracted from cultured cells using TRIzol^®^ Reagent (ThermoFisher, USA). 1 μg mRNA was reverse transcribed with a PrimeScript™ RT reagent Kit with gDNA Eraser (Takara, Dalian, China), and qPCR using FastStart Universal SYBR^®^ Green Master (ROX) (Roche, Germany), qPCR was carried out on an Applied Biosystems 7500 real-time PCR system. For mRNA detection, β-Actin was taken as the endogenous control. The relative levels of mRNA were normalized to controls using the comparative CT method (2^-ΔΔCt^). The primers sequences were listed in **Table 1.**

### Western blot

Cultured cells were lysed with RIPA buffer containing 1% proteinase inhibitor cocktail (Millipore, USA) and PhosSTOP (Roche, Switzerland), followed by centrifuging at 12,000 rpm for 10 min. Separation of cytoplasmic and nuclear protein using PARIS^TM^ Kit (AM1921, Invitrogen^TM^, ThermoFisher, USA). The supernatant protein concentration was measured with a BCA protein assay kit (ThermoFisher, USA), and prepare the cell lyase with 4*LDS loading buffer. Gel electrophoresis was performed on an acrylamide gel and proteins were transferred onto a PVDF membrane (Millipore, USA). Next, the membrane was blocked using 5% BSA and incubated with primary antibody in a shaker at 4 °C overnight. Subsequently, the membrane was rinsed in TBST thrice, each time for 5 min then probed with HRP-conjugated secondary antibody for 2 hours at 4 °C. Finally, blots were detected using chemiluminescent solution and captured.

Antibodies for immunoblotting included anti-NFE2L3 (ABGENT, AP19864B, 1:1000 dilution), anti-N-cadherin (Proteintech, 22018-1-AP, 1:800 dilution), anti-E-cadherin (CST, 3195, 1:1000 dilution), anti-MMP2 (Proteintech, 10373-2-AP, 1:800 dilution), anti-Vimentin (Proteintech, 10366-1-AP, 1:800 dilution), anti-Cyclin D1 (Proteintech, 60186-1-lg, 1:800 dilution), anti-CDK2 (Proteintech, 10122-1-AP, 1:800 dilution), anti-PCNA (Proteintech, 10205-2-AP, 1:800 dilution), anti-Caspase 3 (Proteintech, 19677-1-AP, 1:800 dilution), anti-Caspase 9 (Proteintech, 10380-1-AP, 1:800 dilution), anti-GSK-3β (CST, 9832, 1:1000 dilution), Phospho-GSK-3β (Ser9) (CST, 9322, 1:1000 dilution), anti-β-catenin (CST, 13727, 1:1000 dilution), anti-TCF4 (Proteintech, 22337-1-AP, 1:800 dilution), anti-c-MYC (Proteintech, 10828-1-AP, 1:800 dilution), anti-Histone-H3 (Proteintech, 17168-1-AP, 1:1000 dilution). anti-β-actin (Proteintech, 60008-1-lg, 1:10000 dilution) and anti-GAPDH (Proteintech, 60004-1-lg, 1:10000 dilution) were taken as the endogenous control. Anti-mouse IgG HRP linked Antibody (CST, 7076) and anti-rabbit IgG HRP linked Antibody (CST, 7074) diluted 1:5000. Protein bands were detected with an ECL chemiluminescence reaction kit (Thermo Scientific, USA).

### Statistical analysis

All data were obtained from at least three independent experiments and shown as means±SEM. Graphing and statistical analyses were processed by Origin 8.0 software (OriginLab, USA). For cell experiments, Student's t test (two-tailed) was used to compare differences between groups, One-way analysis of variance (ANOVA), followed by Fisher LSD test, was used for multiple comparisons. Differences were considered to be statistically significant when the *p*-value was less than 0.05.

## Results

### NFE2L3 expression is upregulated in hepatocellular carcinoma tissues and cells

**Figure 1:**
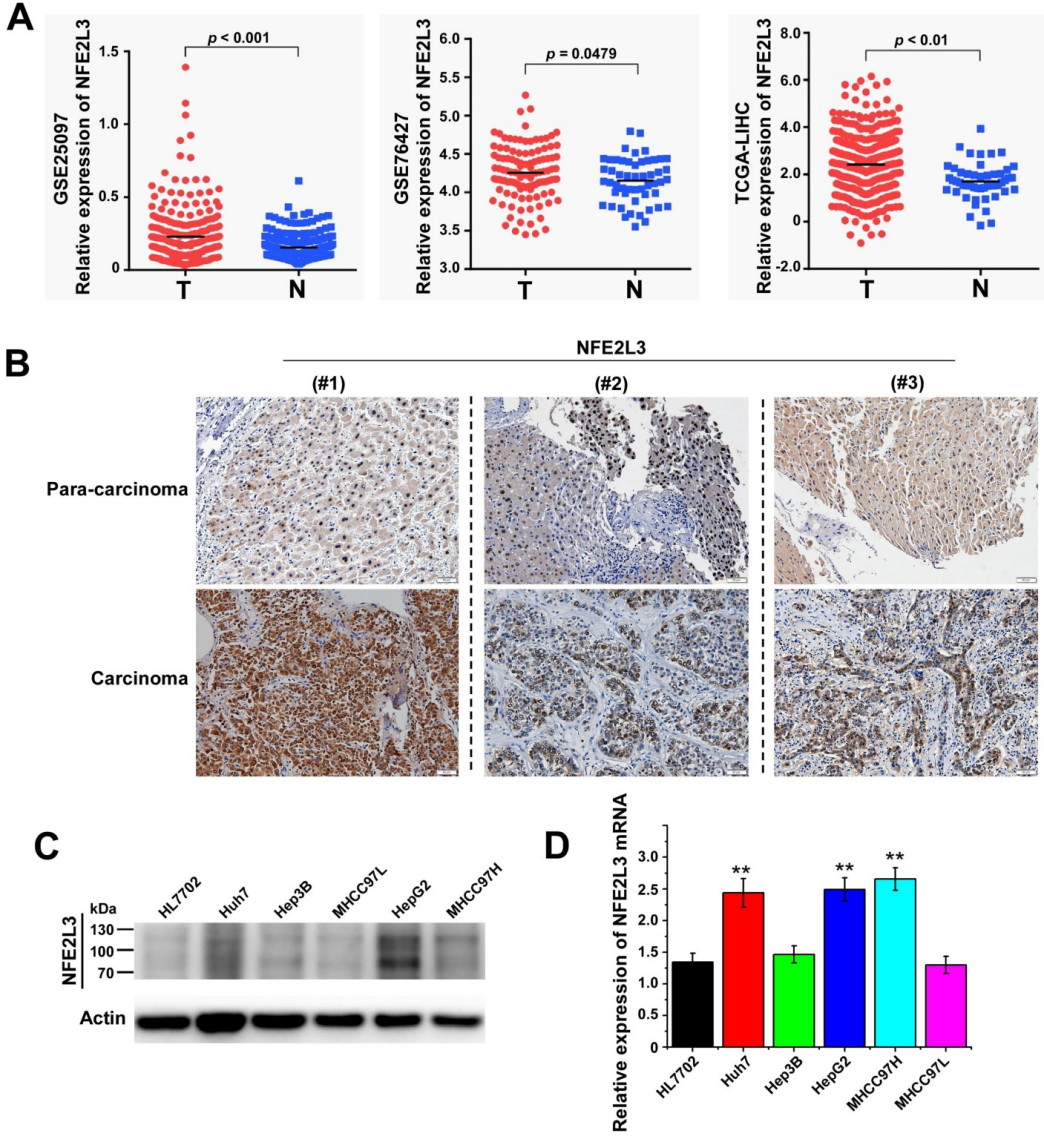
** High expression of NFE2L3 in hepatocellular carcinoma tissues and hepatocellular carcinoma cell lines.** A. NFE2L3 expression level in GEO/TCGA database. The *NFE2L3* mRNA expression level was significantly upregulated in tumor samples (GSE25097: N=243, T=268; GSE76427: N=52; T=115; TCGA: N=51, T=371. N: adjacent non-tumor tissues, T: tumor tissues.). B. Immunohistochemistry result showed that endogenous NFE2L3 expression was increased in carcinoma tissues (n=6) when compared to paired para-carcinoma tissues. C. Western blot. NFE2L3 protein level was higher in hepatocellular carcinoma cells than that in normal liver cell line HL7702. D. RT-qPCR. Expression level of *NFE2L3* mRNA was higher in HepG2 (**p<0.01), Huh7 (**p<0.01) and MHCC97H (**p<0.01) compared to the normal liver cell. All the data from qPCR and western blot were expressed as the mean ± SEM, n=3.

To clarify the expression of NFE2L3 in HCC, we first analyzed RNA-Seq data were obtained from the TCGA/GEO database. According to the analysis results, NFE2L3 expression is markedly elevated in HCC tissues compared with adjacent normal tissues (Fig. 1A). We also detected NFE2L3 by RT-qPCR and western blot in HCC cells, and the results are in good agreement with the prior analysis. The expression levels of NFE2L3's mRNA and protein are both upregulated in HepG2, Huh7 and MHCC97H liver cancer cell lines (Fig. 1C-D). At the same time, the results of IHC also indicated that NFE2L3 was highly expressed in HCC tissues (Fig. 1B). Collectively, these data indicate that NFE2L3 expression is upregulated and associated with HCC.

### Silencing of NFE2L3 decreases cell viability by inhibiting cell proliferation and suppresses the migration of HepG2

**Figure 2:**
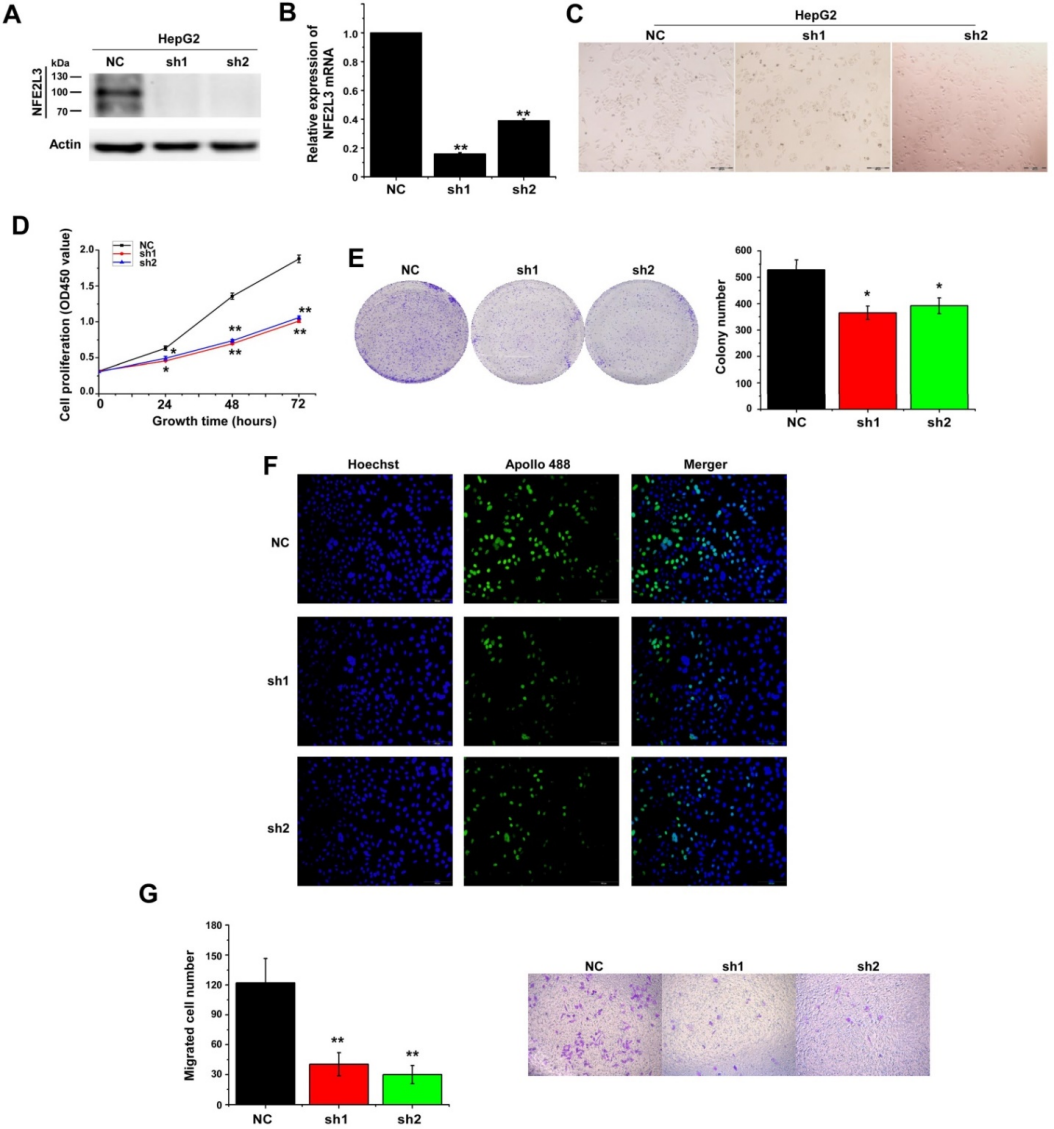
** Silencing of NFE2L3 inhibits cell proliferation and migration of hepatocellular carcinoma cells.** A. Western blot was performed to determine the expression of NFE2L3 in HepG2 cell with transfected NFE2L3 knockdown or scramble sequence lentivirus. β-actin was used as loading control. B. RT-qPCR detected *NFE2L3* mRNA expression in HepG2 cell. *p<0.05, **p<0.01 vs. NC. All error bars represent the SEM from at least 3 independent experiments. C. The above cells were subjected to observation of the morphological changes by optical microscope. D. CCK-8 assay was performed to evaluate the cell viability for different days (1-3 day). *p<0.05, **p<0.01 vs. NC. All error bars represent the SEM from at least 3 independent experiments. E. Colony formation assay was used to assess cell proliferation (left panel) and quantification (right panel) of formation using counting analysis. *p<0.05 vs. NC. All error bars represent the SEM from at least 3 independent experiments. F. Detection of cell proliferation by EdU incorporation assay. G. Cell migration assay was performed to detect the migration ability (left panel) and quantification (right panel) of migrated cells using counting analysis. The cells were stained with crystal violet solution. Image J software was used for cell counting. **p<0.01 vs. NC. All error bars represent the SEM from at least 3 independent experiments. NC: Control (scramble sequence), sh1: NFE2L3 shRNA interference 1, sh2: NFE2L3 shRNA interference 2.

Several studies have reported that decreased NFE2L3 expression inhibits proliferation of cells 15,18. To investigate the potential effect of NFE2L3 on HepG2 cell proliferation, NFE2L3 shRNA interference lentiviral vector was performed to knockdown NFE2L3. Interference efficiency of shRNA was significantly reduced more than 70% in HepG2 compared with the negative control cells (Fig. 2A-B). We first detected cell proliferation by CCK-8, 24 hours after inoculation, the proliferation ability of sh1-NFE2L3 and sh2-NFE2L3 groups were lower than that of the control group (Fig. 2D), which suggested that NFE2L3 played a key role in the proliferation of tumor cells. In plate colony formation experiment, the community formation and proliferation ability of NC group were stronger than that in the sh1 and sh2 groups (Fig. 2E), which was consistent with the results of CCK-8 experiment. In order to understand the effect of NFE2L3 on the proliferation more intuitively, EdU incorporation experiment was used to DNA synthesis detection. Under the fluorescence field, the cell number of green fluorescence decreased in sh1 and sh2 groups, indicating that cells in the proliferative phase decreased and the cellular proliferation capacity was suppressed when NFE2L3 expression was reduced (Fig. 2F). Meanwhile, the migratory ability of the HepG2 in which NFE2L3 deficiency occurred was tested. Transwell analysis showed that knockdown of NFE2L3 significantly inhibited the migration of HepG2 (Fig. 2G). The above results confirm that NFE2L3 produces a critical effect on the migration and metastasis of HCC.

### Down-regulation of NFE2L3 induces cell apoptosis and cell cycle G0/G1 arrest

**Figure 3:**
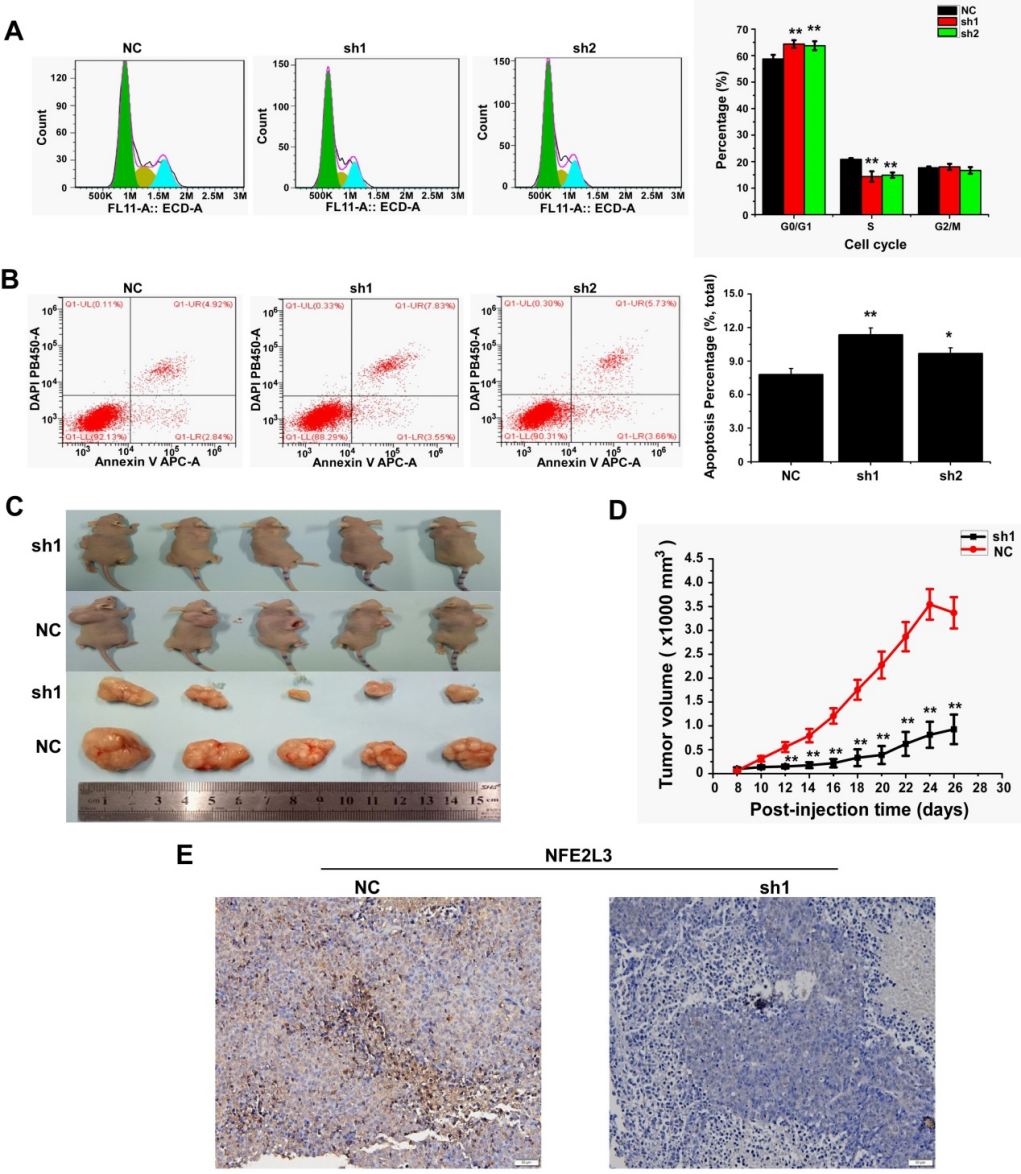
** Knockdown of NFE2L3 induces cell cycle and apoptosis redistribution and inhibits the malignant growth of subcutaneous carcinoma xenograft *in vivo*.** A. Flow cytometry analysis (left panel) of the cell cycle with NFE2L3 knockdown or not. The quantification of the cell cycle distribution (right panel) in the G0/G1 phase, S phase and G2/M phase. **p<0.01 vs. NC. All error bars represent the SEM from at least 3 independent experiments. B. Cell apoptosis was analyzed by flow cytometry (left panel) and the proportion of apoptosis distribution (right panel). *p<0.05, **p<0.01 vs. NC. All error bars represent the SEM from at least 3 independent experiments. C. Shows two groups of different subcutaneous tumor-bearing mice that were inoculated with NC, sh1 cells and different xenograft tumors with different sizes were excised after the mice were sacrificed, and were also subjected to the histopathological examinations (shown below). D. Shows that the tumor sizes were successively measured until four weeks when the mice were sacrificed. The results of growing tumor sizes were calculated as a fold change (mean ± SEM) and then are shown graphically (n=5 per group). **p<0.01 vs. NC. E. Immunohistochemical staining with antibodies against NFE2L3. NC: Control (scramble sequence), sh1: NFE2L3 shRNA interference 1, sh2: NFE2L3 shRNA interference 2.

**Figure 4:**
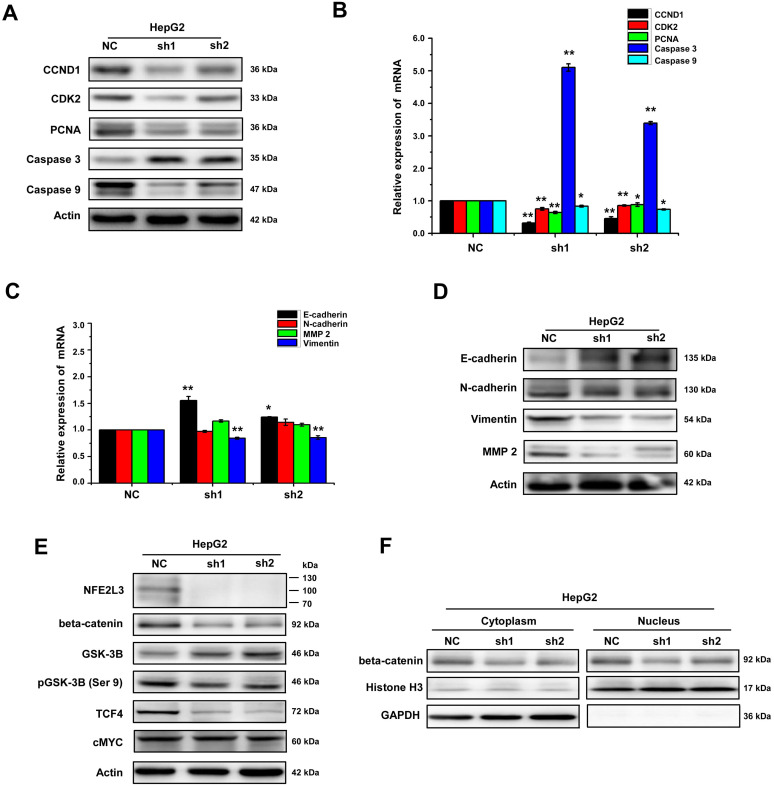
** NFE2L3 promotes EMT via Wnt/β-catenin signaling pathway.** A. The protein expression of genes controlling the cell cycle, apoptosis, metastatic and invasive behavior including the EMT markers were measured by western blot. B. The mRNA expression level of distinct genes controlling cell process and behavior (i.e. cell cycle, apoptosis) were measured by RT-qPCR. (*p<0.05, **p<0.01, vs. NC, n=3). C. The mRNA expression of distinct genes controlling cell metastatic and invasive behavior including the EMT markers were measured by RT-qPCR. (*p<0.05, **p<0.01, vs. NC, n=3). D. Different protein expression level of genes controlling cell metastatic and invasive and EMT markers were measured by western blot. E. Representative blots of β-catenin, GSK3-β, pGSK-3β (Ser9), c-MYC and TCF4. F. Representative blots of cytoplasmic and nuclear β-catenin, All error bars represent the SEM. NC: Control (scramble sequence), sh1: NFE2L3 shRNA interference 1, sh2: NFE2L3 shRNA interference 2.

We detected cell cycle and cell apoptosis using flow cytometry. As shown in the results, down-regulation of NFE2L3 resulted in cell cycle arrest at G0/G1 phase; the S phase of the sh1 and sh2 groups was reduced remarkably in comparison to the NC group (Fig. 3A). Moreover, silencing NFE2L3 increases cell apoptosis induced by hydrogen peroxide (Fig. 3B). For a molecular mechanism of the above changes in cell cycle and apoptosis, RT-qPCR and western blot were performed to examine the crucial genes. As expected, the results showed that knockdown of NFE2L3 activated Caspase 3, and decreased expression of Caspase 9, Cyclin D1, CDK2 and PCNA (Fig. 4A-B). This is also consistent with the published conclusions.

### Deficiency of NFE2L3 inhibits the malignant growth of subcutaneous carcinoma xenograft *in vivo*

Knockdown of NFE2L3 has been proved to inhibit the malignant behavior of tumor cells *in vitro*. Since anchorage-independent growth is a hallmark of tumorigenesis 19, an animal xenograft model was next used to test the effect of NFE2L3 deficiency elicits on the carcinogenesis of tumor cells. After either HepG2 cells (NC, sh1) were inoculated subcutaneously into the back-right region of nude mice. These tumor xenografts had been clearly seen until eight days after subcutaneous inoculation. The tumor sizes were measured at one-day intervals, and results were calculated as showed graphically. The tumor growth curve displays that NFE2L3 deficient carcinoma xenografts were slowly growing at a steady rate (Fig. 3D). By contrast, the NC cells-derived xenografts followed by a rapid growth and they were expanding exponentially in size until day 26 (Fig. 3C-D). Further IHC revealed that NFE2L3 in sh1-derived xenograft tissues have marked reduction (Fig. 3E). This observation suggests that loss of NFE2L3 inhibits the malignant growth of subcutaneous carcinoma xenograft in nude mice.

### NFE2L3 promotes EMT via activating Wnt/β-catenin pathway during tumorigenesis and progression

Collectively, the aforementioned results demonstrate that loss of NFE2L3 leads to significantly change in cell cycle and malignant behavior, such as transformation, migration, tumourigenesis and overgrowth of the carcinoma xenografts derived from HepG2 cells. Moreover, in the process of cell culture, it was observed obviously that the morphology of the HepG2 cells changed from mesenchymal to epithelial appearance with NFE2L3 stable knockdown (Fig. 2C). It was hypothesized that high level of NFE2L3 may trigger EMT. E-cadherin, N-cadherin, Vimentin and β-catenin have been frequently identified as specific biomarkers for the EMT process 20,21. Therefore, RT-qPCR and western blot were performed to determine whether NFE2L3 had an effect on the constitutive expression of EMT markers. As presented in Fig. 4C, the mRNA levels of epithelial marker E-cadherin was significantly increased in silencing NFE2L3 cells, whereas mesenchymal marker Vimentin was significantly decreased and N-cadherin was not affected. Western blot analysis revealed that β-catenin; Vimentin and N-cadherin were markedly downregulated in sh1 and sh2 cells (Fig. 4D). In addition, expression of MMP-2 was dysregulated in sh1 and sh2 (Fig. 4D), it was hence postulated to increase the breakdown of extracellular matrix proteins insomuch as to induce cancer cell metastasis. These results thus indicate that the silencing of NFE2L3 attenuates EMT in HCC.

Evidence has shown that Wnt/β-catenin pathway plays an essential role in promoting tumorigenesis particularly invasion, metastasis and malignant proliferation 22. It was not distinct whether NFE2L3 promotes the progression of HCC via the Wnt/β-catenin signaling pathway. For this reason, we investigated the effect of NFE2L3 on Wnt/β-catenin pathway by detecting expression of β-catenin and Wnt target genes including cyclin D1, c-MYC and TCF4 in both NC, sh1 and sh2 cells. As shown in Fig. 4E, silencing of NFE2L3 significantly reduced the protein levels of β-catenin, cyclin D1 and TCF4. At the same time, the protein expression of β-catenin was decreased at both cytoplasm and nucleus (Fig. 4F). Furthermore, we also detected the expression and phosphorylation level of GSK-3β, which upstream of β-catenin. Western blot results showed that knockdown NFE2L3 upregulated the expression of total GSK-3β and downregulated the phosphorylation level of GSK-3β at Ser9 (Fig. 4E). Similarly, down-regulation of NFE2L3 also inhibited expression of Wnt target genes such as cyclin D1, CDK2 and PCNA (Fig. 4A-B), that confirming its effect on Wnt/β-catenin pathway. Taken together, these data suggested that NFE2L3 promoting the EMT in HCC might be associated with its activation effect on Wnt/β-catenin signaling pathway.

## Discussion

Surgical resection of hepatocellular carcinoma with early diagnosis results in a high five-year survival rate. However, metastasis had already occurred at the time of diagnosis in most patients, thus early diagnosis and treatment is of great importance. The occurrence and development of cancers involve multiple impacts such as transcriptional regulation, epigenetics and environment. Genes expression is differentiated in many cancers, and is closely related to tumor development and progression, including liver cancer 23,24.

It is well known that oxidative stress is bound up with tumorigenesis in many cancer types 25,26. The Cap 'n' Collar (CNC) family plays a vital role in the regulation of oxidant stress and tumorigenesis 27. As the most concerned research member, hundreds of publications have well defined functions of NFE2L2 (NRF2) and NFE2L1 (NRF1) in multiple cancers 28-30. However, few articles have involved the functions of its homologous gene NFE2L3 in cancer development especially in hepatocellular carcinoma.

Gene expression profiling based on large data sets plays an increasingly important role in exploring new potential tumor molecular markers 31. In this study, combined with analyzing the TCGA/GEO database, data analysis results state clearly that NFE2L3 mRNA expression remarkably elevated in HCC as compared with para-cancerous tissues. IHC showed that NFE2L3 protein expression was also increased in hepatocellular carcinoma tissues. Moreover, by comparing the expression of NFE2L3 in five human liver cancer cell lines, the data suggested there was an up-regulation of NFE2L3 in cancer cells, while there was lower-expression in normal ones. These findings implied NFE2L3 might be a cancer-promoting gene in human liver cancer, which was contrary to the protective effect of NFE2L3 on lymphomagenesis 13. Subsequently, loss function experiments revealed that knockdown NFE2L3 inhibits the cell proliferation by increasing the cell number in the G0/G1 phase, while reducing the cell number in the S phase. Nevertheless, we found that the expression of NFE2L3 in Hep3B and MHCC97L didn't show the difference between the control groups. The difference of genetic background may lead to the differential expression of NFE2L3; meanwhile, the human liver cancer cell lines established *in vitro* will undergo a transformation, including gene loss, drift, etc. More importantly, tumor cells are polymorphic, and the differential expression of NFE2L3 is the cause of tumor heterogeneity.

Furthermore, knockdown of NFE2L3 significantly impairs both migration ability in HepG2 cells, which was consistent with the data that NFE2L3 is positive correlation with malignant metastasis status of cancer. Metastatic tumor cells should first alter cell adhesion. EMT, a process acting as pivotal mechanism in the invasiveness drive in most cases of cancer 32, which determined to be the formation of a three-layer embryo during early embryonic development 33,34. During EMT, the process begins with the disintegration of cell adhesion, increased motility, and chemotherapeutic resistance by losing epithelial markers, such as E-cadherin, and expressing mesenchymal markers, such as N-cadherin and Vimentin 35. Therefore, we tried to determine the role of NFE2L3 in the EMT of liver cancer cells. Significantly, downregulation NFE2L3 remarkably inhibited the EMT of HepG2 cell by upregulating the expression of E-cadherin and downregulating the expression of N-cadherin and Vimentin. Meanwhile, we also found downregulated NFE2L3 will inhibit the expression of MMP-2. Thus all these data reveal that NFE2L3 could be instrumental in tumor migration by promoting both EMT process and the degradation of ECM.

Wnt/β-catenin pathway plays an indispensable role in development as well as homeostasis of organisms. However, deviant activation of the Wnt/β-catenin pathway is highly correlated with oncogenesis. When activated, expression of downstream target genes such as cyclin D1 and c-MYC are upregulated, leading to promotion of tumor cell proliferation, invasion and migration 36. Meanwhile, researches have suggested that Wnt/β-catenin signaling pathway promotes EMT 37. Therefore, we try to explore the correlation between NFE2L3 and Wnt/β-catenin signaling pathway in HCC, we found that silencing of NFE2L3 decreased the expression of β-catenin and the target genes TCF4 and cyclin D1. Consistent with the above results, the expression of CDK2 and PCNA also decreased, it is further confirmed that NFE2L3 plays an important role in cell growth and proliferation. Cyclin D1 is a key mediator of cell growth and proliferation, some researches had proved that NFE2L3 regulated the expression of cyclin D1 in CRC while another article pointed out that the silencing of NFE2L3 will increase the level of DUX4, a direct inhibitor of CDK1, and reduce the proliferation and tumorigenicity of colon cancer cells 15,18. In addition, both cytoplasmic and nuclear β-catenin were decreased by knockdown NFE2L3, indicating NFE2L3 significantly regulated transcription of β-catenin in liver cancer cells. Furthermore, we also detected the expression and phosphorylation level of GSK-3β, which upstream of β-catenin. Results showed that knockdown NFE2L3 upregulated the expression of total GSK-3β and downregulated the phosphorylation level of GSK-3β at Ser9. Therefore, we speculate that NFE2L3 promotes EMT in HCC via activation of Wnt/β-catenin signaling pathway.

## Conclusion

In conclusion, our research study demonstrates that NFE2L3 promotes cell proliferation and metastasis, accompanied by enhancement of EMT *in vitro*. Meanwhile, silencing of NFE2L3 also shows an inhibitory effect on tumor growth in HepG2 cell xenografts *in vivo*. And NFE2L3 may play a role of oncogene by activating Wnt/β-catenin signaling pathway.

## Figures and Tables

**Table 1:** Primers for qPCR

Primer name	Source	Nucleotide sequences (5′ to 3′)
NFE2L3 FW	Sangon	CACAGATAGAAACTTGAGCCGT
NFE2L3 REW	Sangon	GCGTTTACGACAGTTCTGCG
CCND1 FW	Sangon	GCTGCGAAGTGGAAACCATC
CCND1 REW	Sangon	CCTCCTTCTGCACACATTTGAA
PCNA FW	Sangon	GCGTGAACCTCACCAGTATGT
PCNA REW	Sangon	TCTTCGGCCCTTAGTGTAATGAT
CDK2 FW	Sangon	CCAGGAGTTACTTCTATGCCTGA
CDK2 REW	Sangon	TTCATCCAGGGGAGGTACAAC
Caspase 3 FW	Sangon	CATGGAAGCGAATCAATGGACT
Caspase 3 REW	Sangon	CTGTACCAGACCGAGATGTCA
Caspase 9 FW	Sangon	CTCAGACCAGAGATTCGCAAAC
Caspase 9 REW	Sangon	GCATTTCCCCTCAAACTCTCAA
MMP2 FW	Sangon	TACAGGATCATTGGCTACACACC
MMP2 REW	Sangon	GGTCACATCGCTCCAGACT
Vimentin FW	Sangon	GACGCCATCAACACCGAGTT
Vimentin REW	Sangon	CTTTGTCGTTGGTTAGCTGGT
E-cadherin FW	Sangon	CGAGAGCTACACGTTCACGG
E-cadherin REW	Sangon	GGGTGTCGAGGGAAAAATAGG
N-cadherin FW	Sangon	TCAGGCGTCTGTAGAGGCTT
N-cadherin REW	Sangon	ATGCACATCCTTCGATAAGACTG
β-actin FW	Sangon	CATGTACGTTGCTATCCAGGC
β-actin REW	Sangon	CTCCTTAATGTCACGCACGAT

## References

[1] An L, Zeng HM, Zheng RS (2019). Liver cancer epidemiology in China, 2015. Zhong Hua Zhong Liu Za Zhi.

[2] Siegel RL, Miller KD, Jemal A (2020). Cancer statistics, 2020. CA Cancer J Clin.

[3] Sarveazad A, Agah S, Babahajian A (2019). Predictors of 5 year survival rate in hepatocellular carcinoma patients. J Res Med. Sci.

[4] Fujiwara N, Friedman SL, Goossens N (2018). Risk factors and prevention of hepatocellular carcinoma in the era of precision medicine. J Hepatol.

[5] Gounder MM, Mahoney MR, Van Tine BA (2018). Sorafenib for Advanced and Refractory Desmoid Tumors. N Engl J Med.

[6] Kobayashi A, Ito E, Toki T (1999). Molecular cloning and functional characterization of a new Cap 'n' collar family transcription factor Nrf3. J Biol Chem.

[7] Liu P, Kerins MJ, Tian W (2019). Differential and overlapping targets of the transcriptional regulators NRF1, NRF2, and NRF3 in human cells. J Biol Chem.

[8] Chevillard G, Blank V (2011). NFE2L3 (NRF3): the Cinderella of the Cap 'n' Collar transcription factors. Cell Mol Life Sci.

[9] Kobayashi A, Waku T (2020). New addiction to the NRF2-related factor NRF3 in cancer cells: Ubiquitin-independent proteolysis through the 20S proteasome. Cancer Sci.

[10] Baird L, Yamamoto M (2020). The Molecular Mechanisms Regulating the KEAP1-NRF2 Pathway. Mol Cell Biol.

[11] Waku T, Nakamura N, Koji M (2020). NRF3-POMP-20S Proteasome Assembly Axis Promotes Cancer Development via Ubiquitin-independent Proteolysis of p53 and Retinoblastoma Protein. Mol Cell Biol.

[12] Aono S, Hatanaka A, Gao Y (2019). β-Catenin/TCF4 complex-mediated induction of the NRF3 (NFE2L3) Gene in Cancer Cells. Int J Mol Sci.

[13] Chevillard G, Paquet M, Blank V (2011). Nfe213 (Nrf3) deficiency predisposes mice to T-cell lymphoblastic lymphoma. Blood.

[14] Sun J, Zheng Z, Chen Q (2019). NRF3 suppresses breast cancer cell metastasis and cell proliferation and is a favorable predictor of survival in breast cancer. Onco Targets Ther.

[15] Zhang L, Hu DL, Tang B (2019). NFE2L3 Inhibition Induces Cell Cycle Arrest at the G0/G1 Phase in Colorectal Cancer Cells through Downregulating CCND1 and pRb1-ser807/811. Dis Markers.

[16] McCarthy DJ, Chen Y, Smyth GK (2012). Differential expression analysis of multifactor RNA-Seq experiments with respect to biological variation. Nucleic Acids Res.

[17] Morton CL, Houghton PJ (2007). Establishment of human tumor xenografts in immunodeficient mice. Nat Protoc.

[18] Bury M, Le Calvé B, Lessard F (2019). NFE2L3 Controls Colon Cancer Cell Growth through Regulation of DUX4, a CDK1 Inhibitor. Cell Rep.

[19] Dong Z, Cmarik JL (2002). Harvesting cells under anchorage-independent cell transformation conditions for biochemical analyses. Sci STKE.

[20] Hu X, Zhai Y, Kong P (2017). FAT1 prevents epithelial mesenchymal transition (EMT) via MAPK/ERK signaling pathway in esophageal squamous cell cancer. Cancer Lett.

[21] Chen HA, Kuo TC, Tseng CF (2016). Angiopoietin-like protein 1 antagonizes MET receptor activity to repress sorafenib resistance and cancer stemness in hepatocellular carcinoma. Hepatology.

[22] Ren Y, Tao J, Jiang Z (2018). Pimozide suppresses colorectal cancer via inhibition of Wnt/β-catenin signaling pathway. Life Sci.

[23] Reiter F, Wienerroither S, Stark A (2017). Combinatorial function of transcription factors and cofactors. Curr Opin Genet Dev.

[24] Mateo L, Guitart-Pla O, Duran-Frigola M (2018). Exploring the OncoGenomic landscape of cancer. Genome Med.

[25] Chio IIC, Tuveson DA (2017). ROS in Cancer: The Burning Question. Trends Mol Med.

[26] Liou GY, Storz P (2010). Reactive oxygen species in cancer. Free Radic Res.

[27] Gęgotek A, Skrzydlewska E (2015). CNC proteins in physiology and pathology. Postepy HigMed Dosw (Online).

[28] Taguchi K, Motohashi H, Yamamoto M (2011). Molecular mechanisms of the Keap1-Nrf2 pathway in stress response and cancer evolution. Genes Cells.

[29] Ren Y, Qiu L, Lü FL (2016). TALENs-directed knockout of the full-length transcription factor Nrf1α that represses malignant behaviour of human hepatocellular carcinoma (HepG2) cells. Sci Rep.

[30] Xu D, Xu M, Jeong S (2019). The role of Nrf2 in Liver Disease: Novel molecular mechanisms and therapeutic approaches. Front Pharmacol.

[31] Shahjaman M, Kumar N, Ahmed MS (2017). Robust feature selection approach for patient classification using gene expression data. Bioinformation.

[32] Gurzu S, Silveanu C, Fetyko A (2016). Systematic review of the old and new concepts in the epithelial-mesenchymal transition of colorectal cancer. World J Gastroenterol.

[33] Puisieux A, Brabletz T, Caramel J (2014). Oncogtenic roles of EMT-inducing transcription factors. Nat Cell Biol.

[34] Van Staalduinen J, Baker D, Ten Dijke P (2018). Epithelial-mesenchymal-transition-inducing transcription factors: new targets for tackling chemoresistance in cancer?. Oncogene.

[35] Peng JM, Bera R, Chiou CY (2018). Actin cytoskeleton remodeling drives epithelial-mesenchymal transition for hepatoma invasion and metastasis in mice. Hepatology.

[36] Xiang D, Cheng Z, Liu H (2017). Shp2 promotes liver cancer stem cell expansion by augmenting β-catenin signaling and predicts chemotherapeutic response of patients. Hepatology.

[37] Chai S, Ng KY, Tong M (2016). Octamer 4/microRNA-1246 signaling axis drives Wnt/β-catenin activation in liver cancer stem cells. Hepatology.

